# Grip strength measured by high precision dynamometry in healthy subjects from 5 to 80 years

**DOI:** 10.1186/s12891-015-0612-4

**Published:** 2015-06-10

**Authors:** Jean-Yves Hogrel

**Affiliations:** Institut de Myologie, GH Pitié-Salpêtrière, 75651 Paris Cedex 13, France

**Keywords:** Muscle strength, Dynamometry, Handgrip, Normative data, Prediction model, Outcome measures

## Abstract

**Background:**

Grip strength is a variable which may be important to measure and follow in various populations. A new dynamometer with high accuracy and sensitivity has recently been developed to assess grip strength. The objectives of this work were to provide norms of maximal isometric grip strength measured with this new dynamometer (the MyoGrip device), to assess the reliability of measurements, to compare the measurements obtained with MyoGrip and Jamar dynamometers and finally to establish predictive equations from a population of healthy subjects (children and adults).

**Methods:**

Measurements of maximal isometric grip strength using the MyoGrip and the Jamar (which is considered as the gold-standard) were performed on 346 healthy subjects aged from 5 to 80 years. Test-retest reliability for both devices was assessed on 77 subjects. Predictive equations were computed on subjects younger than 60 years of age in order to avoid the effects of aging on strength.

**Results:**

This study provides norms for isometric grip strength for health subjects from 5 to 80 years. Reliability of the MyoGrip device was excellent (intraclass correlation coefficient: 0.967). Despite good correlation between devices, the Jamar tended to overestimate maximal grip strength by about 14 %. A single predictive equation for men and women, adults and children incorporating hand circumference only can be used to compute the predicted theoretical maximal grip strength.

**Conclusions:**

The MyoGrip device is a reliable tool for measuring isometric grip strength. Owing to its unique metrological features, it can be used in very weak patients or in any situation where high precision and accuracy are required.

## Background

Over the past 15 years extensive literature has described a relationship between grip strength and various functional, clinical, psychological or psychosocial parameters in different populations, particularly in elderly people [1]. Measurement of maximal grip strength (MGS) is an essential element to follow people during growth, aging, injury, rehabilitation, training or therapeutic trials. Its measurement is performed using dynamometers, which estimate the muscle strength primarily generated by the flexor muscles of the hand and the forearm. Different types of dynamometers are available, with such devices classified as hydraulic, pneumatic, mechanical and electronic [2]. These dynamometers vary in terms of their mechanism, performance, display mode and energy supply.

The Jamar dynamometer is the most widely reported device used to measure grip strength [3–10]. Eighty percent of occupational therapy schools and clinics in the United States use the Jamar dynamometer as their usual instrument to assess grip strength [2]. The Jamar has many useful features for routine screening as well as in the evaluation of hand trauma and disease. The Jamar displays grip force in both pounds and kilograms, with a maximum of 200 lb (90 kg). It has a peak-hold needle that automatically retains the highest reading until reset. The Jamar test is isometric, with no perceptible motion of the handle, regardless of the grip strength applied. The handle can be adjusted for different size in order to fit for individual use. The Jamar dynamometer presents good inter-rater reliability and test-retest reliability [11]. The American Society of Hand Therapists (ASHT) has recommended the Jamar dynamometer as the gold standard, leading to its widespread use in clinical practice and research [12].

However, the Jamar "may not be the most appropriate for all patient populations" [13]. This instrument is not consistent for use in people with weak strength and the resolution of the Jamar is too large to detect small changes in strength [14]. Massy-Westropp et al. [15] observed that 18 of their subjects (representing nearly 15 % of 121 patients tested) could not be assessed by the Jamar, but their grip strength was detectable by a digital device (Grippit). Still, for very weak people, a reliable dynamometer was required. This was the reason for developing the MyoGrip, an innovative dynamometer presenting high metrological performances.

The objectives of this study were (1) to validate an innovative hand grip dynamometer from a metrological point of view, (2) to establish normative data for grip strength using this dynamometer, (3) to assess the reliability of measurements, (4) to compare the results with those of the Jamar as a "gold-standard", and (5) to establish predictive equations for MGS from a population of healthy subjects.

## Methods

### Participants

Healthy subjects, male and female, aged from 5 to 80 years old were recruited by advertisements in newspapers, websites, and posters. Exclusion criteria were any neurological, neuromuscular or other disorders that could affect muscle strength, any history of injury, disease, pain or discomfort involving the upper limbs in the last two years, and practice of a sport at a national level. Subjects were informed about the terms of the experimental protocol and procedures before giving their written consent. The protocol (namely MyoTools) was approved by the Local Ethics Committee (CPP-Ile de France VI) and aimed to assess muscle strength in several muscle functions (hand grip, wrist extension and flexion, ankle flexion and extension). All subjects gave written informed consent to participate in the measurement sessions.

### Anthropometric measurements

**Fig. 1:**
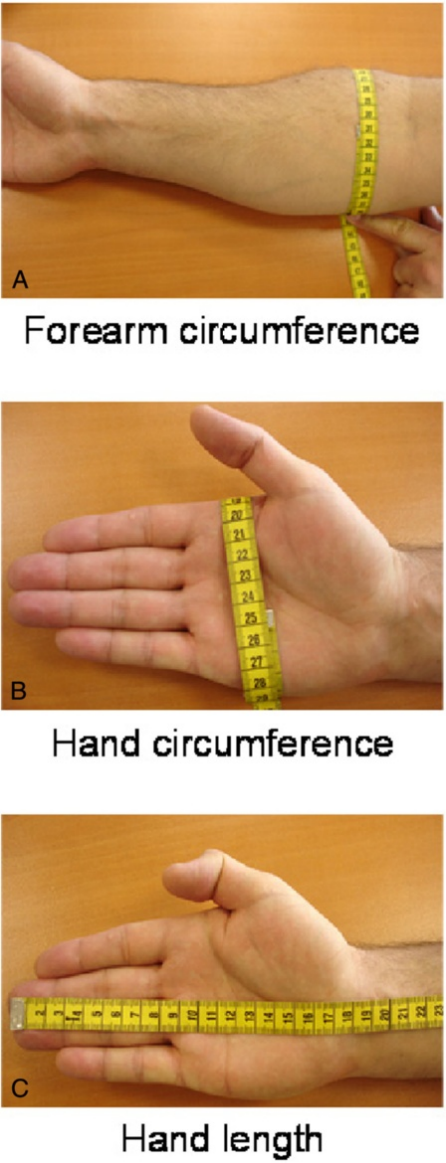
Measurements of anthropometric characteristics of hand and forearm including forearm circumference (**a**), hand circumference (**b**) and hand length (**c**). The measurements were performed to the nearest millimeter by means of a supple tape measure. Strict anatomical landmarks were used to correctly position the tape

The height and weight of the subjects were recorded as well as an estimation of the percentage of body fat mass using an impedance metric scale (Tanita TBF-543). Anthropometric hand data were measured by the experimenter using a standard 1000-mm tape measure. The circumference of the forearm was defined as the perimeter of the largest part of the forearm, located over the bulk of the brachioradialis muscle, at the proximal quarter of the whole forearm length (Fig. 1a). The circumference of the hand (C_hand_) was measured as the perimeter of the middle part of hand, located at the two major transverse palmar creases ("heart line" and "head line") (Fig. 1b). Hand length was defined as the distance from the tip of the middle finger to the midline of the distal wrist crease (Fig. 1c). All anthropometric data were measured to the nearest millimetre with the forearm and hand in an outstretched and supinated position. Dominant side was defined as the hand with which the subject writes.

### Dynamometer description

**Fig. 2:**
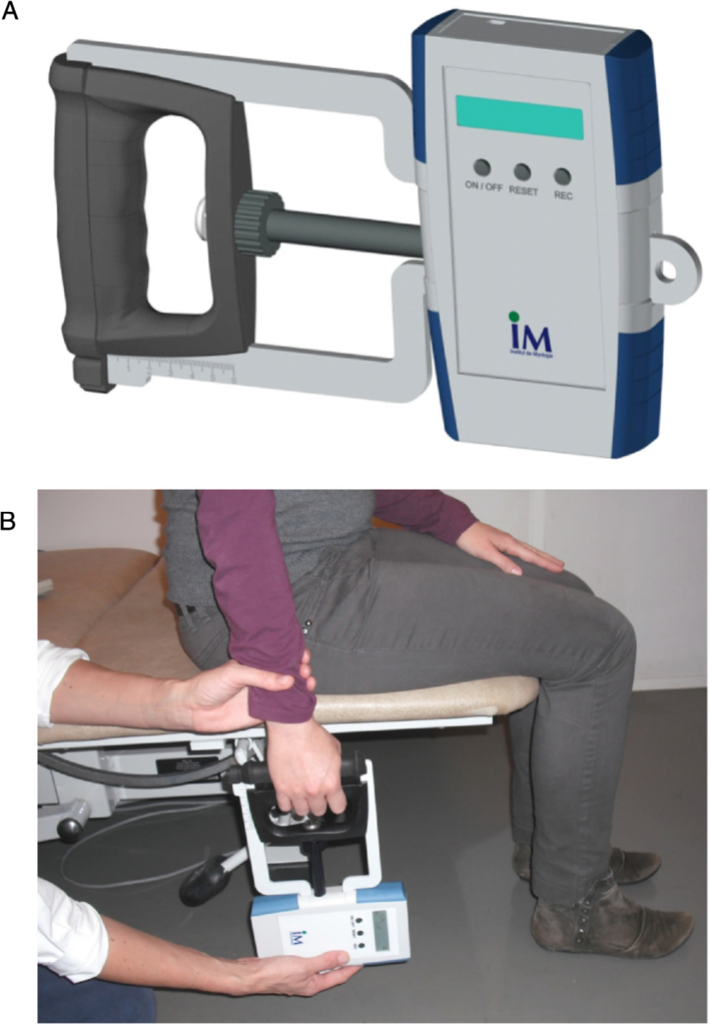
MyoGrip dynamometer (**a**) and measurement positioning (**b**). The evaluator maintained the wrist of the subject to control possible compensatory movements

The Myogrip dynamometer (Ateliers Laumonier, France) is an isometric electronic device specifically developed for measuring grip strength in weak patients (Fig. 2a). It can directly display strength on its screen or be connected to a computer either by wireless, RS232 or BNC connections. Handle size is adjustable in a continuous way. It measures force in kg. It is calibrated on consecutive linear segments to compensate possible non-linear behaviour on the full nominal scale (90 kg). The resulting accuracy reaches 50 g on the whole measurement range with a 10 g resolution. To the best of our knowledge, the metrological performances of this innovative dynamometer are unique.

### Calibration quality control

Devices were checked using standardized operating procedures for accuracy, hysteresis and repeatability. The procedure was adapted from the ISO 17025 norm. Twelve weights using M3 class masses were used for checking the calibration (0.2, 0.5, 1, 1.5, 2, 4, 5, 8, 10, 20, 30, 50 kg). Six MyoGrip devices and two Jamar devices were checked for calibration using this procedure. The devices were suspended to a bracket and the masses were directly applied on the handle. The Jamar was unable to accurately detect forces below 5 kg.

### Experimental procedure

The subjects were seated on a height-adjustable plinth in order to obtain a right angle at the hip, knee and ankle joints with the legs being vertical and feet flat on the ground (Fig. 2b). The subjects had their shoulders adducted and their testing arm close to their body, with their elbow in full extension.

Subjects were verbally encouraged to produce their maximal grip strength (MGS). Two trials were first recorded, consisting of a 2-4-second maximal contraction, with a 30-second rest period between each trial. If the relative difference between these two MGS was within 10 %, no additional trial was required. If not, additional trials were proposed until two reproducible MGS were obtained. The maximal value of the two reproducible trials was retained for analyses. The contralateral side was then tested according to the same procedure. The first tested side (i.e., right or left) was random.

A subgroup of subjects agreed to return to retest their grip strength. The experimental conditions were the same as in the first session. The evaluator was either the same or another evaluator trained in the experimental procedures. Three evaluators performed the measurements for reliability assessment. The retest session was performed at least one day after the first session or planned within the next 3 months (mean: 31 days).

Jamar and Myogrip devices were checked for calibration before the recording period.

### Statistical analyses

Norms were established in kg by age group categories of five years for younger subjects up to 20 years old and then by age groups of 10 years. In order to decide whether norms should be established according to the side tested or to the dominant side, the MGS values between the right and left sides were compared taking into account the dominance effect. The MGS values between the dominant and non-dominant sides in both right-handed and left-handed groups were compared by means of a paired Student *t*-test.

The difference between test and retest sessions was evaluated by taking into account the rater effect and the side effect for each function using a repeated measurements analysis of variance. The standard error of measurement, the coefficient of variation (CVar) and the limits of agreement according to Bland and Altman [16] were calculated. Correlation between MGS obtained by the MyoGrip and the Jamar dynamometers was tested using a correlation analysis (Pearson). To assess reliability within and between dynamometers, the intra-class correlation coefficient (ICC_2,1_) was computed as a single measure ICC with a two-way random-effect model (absolute agreement). The ability of the device to discriminate between two measurements was computed as the smallest detectable difference (SDD) according to Beckerman et al. [17]. The agreement between dynamometers was also studied using Bland & Altman plots.

Predictive analyses were performed only on the subjects aged less than 60 to avoid the influence of aging on the model. Indeed, according to norms (e.g., [18]) and functional studies (e.g., [19]), dynapenia becomes significant and accelerates after the age of 60. Stepwise linear regressions were performed to detect the best predicting variables for MGS. Variables tested were height, weight, age, sex, body mass index, percentage of body fat, hand circumference, hand length and forearm circumference. Since hand circumference was found to be the best variable in explaining inter-individual variance, various models using this variable alone were tested to define the best one in terms of explained variance. Predictive equations were applied to the subjects to compute predicted strength values. The statistical analyses were performed using SPSS (v19.0). The limit of significance for all tests was set at p < 0.05.

## Results

### Subjects

Three hundred and forty six subjects were evaluated: 58 children under 18 years (29 boys, 29 girls) and 288 adults (119 men, 169 women). Their main characteristics are presented in Table 1; 9.5 % of the subjects were left-handed. Interestingly, the circumference of the left hand was significantly smaller than the circumference of the right hand (mean difference: 2.0 ± 4.3 mm; p < 0.0001). The measurements took approximately 20 min to be performed on both sides using the two dynamometers. There were no adverse events nor discomfort during testing.

**Table 1:** Main characteristics of the experimental population

Age range (years)	Gender	Number	Age (years)		Height (cm)		Weight (kg)		BMI (kg/m^2^)		% fat mass		Hand circumference (cm)			
													left		right	
			mean	SD	mean	SD	mean	SD	mean	SD	mean	SD	mean	SD	mean	SD
5–10	F	12	8.0	1.4	127.8	10.4	28.3	7.8	17.0	1.8	23.9	4.4	15.5	1.1	15.6	1.0
	M	15	6.4	1.2	118.0	8.3	22.8	4.6	16.2	1.4	14.1	4.7	15.2	0.7	15.2	0.7
10–15	F	9	12.4	1.4	154.1	11.2	44.1	11.5	18.3	2.5	23.6	3.8	17.4	0.7	17.7	0.8
	M	11	12.3	1.4	155.5	10.4	44.8	9.9	18.3	2.1	16.7	4.4	18.4	1.3	18.5	1.1
15–20	F	15	17.5	1.5	164.6	5.0	59.4	11.4	21.9	3.9	26.2	6.0	18.5	0.8	18.6	0.9
	M	10	18.1	1.5	182.0	6.7	74.9	15.8	22.4	3.4	15.4	7.0	21.2	1.2	21.4	1.3
20–30	F	32	25.6	2.9	167.2	6.7	64.4	15.9	22.9	5.0	29.6	6.8	18.9	0.9	18.9	1.0
	M	27	24.5	2.9	177.8	4.9	74.9	10.3	23.7	3.5	17.3	5.7	21.2	0.8	21.6	0.8
30–40	F	31	35.0	2.8	164.5	5.8	62.8	10.1	23.2	3.6	28.5	7.8	19.0	1.0	19.3	1.1
	M	32	35.4	3.0	176.5	6.6	76.4	12.9	24.5	3.6	18.7	7.2	21.4	0.9	21.6	1.0
40–50	F	32	45.3	3.1	163.8	5.0	62.4	8.9	23.3	3.5	28.5	8.2	18.8	0.7	19.2	0.9
	M	26	44.5	3.1	176.4	6.1	77.3	12.9	24.7	3.3	17.9	5.5	22.0	1.0	22.3	1.2
50–60	F	29	55.1	2.5	162.2	6.1	63.9	10.7	24.4	4.5	29.8	7.5	19.4	0.8	19.5	0.9
	M	11	54.0	3.4	178.3	7.4	78.8	10.2	24.8	2.4	19.0	4.5	22.1	0.6	22.5	0.6
60–70	F	21	64.8	2.9	160.2	7.3	62.8	10.6	24.4	3.4	29.8	6.7	19.3	0.9	19.4	0.8
	M	11	64.4	3.5	172.7	6.8	84.7	13.0	28.2	2.7	24.2	5.3	22.1	1.0	22.1	1.1
70–80	F	17	73.7	2.8	161.3	5.0	62.8	8.1	24.2	3.3	28.8	7.7	19.6	0.8	19.8	0.7
	M	5	74.5	2.9	173.2	5.0	84.3	13.1	28.0	3.1	22.8	5.8	22.0	0.7	21.9	0.4

### Assessment of metrological properties

All of the MyoGrip devices were tested using a standardized calibration procedure which showed that the device was 50 g accurate over the whole measuring range (from 0 to 90 kg) with a 10 g sensitivity. No hysteresis was detectable and reproducibility was almost perfect (always less than 50 g difference between calibration trials).

### Normative data

The right-handed subjects were significantly stronger on their dominant side (mean difference: 2.3 ± 3.7 kg; p < 0.0001); this was not the case for the left-handed subjects (mean difference: 0.6 ± 5.1 kg; p = 0.775). The data are thus presented as left and right sides rather than dominant and non-dominant sides (Table 2).

**Table 2:** Mean muscle strength and standard deviation of hand grip according to age, gender and side for MyoGrip and Jamar dynamometers

Age range (years)	Gender	Number	Age (years)		Left MGS (kg)				Right MGS (kg)			
					MyoGrip		Jamar		MyoGrip		Jamar	
			mean	SD	mean	SD	mean	SD	mean	SD	mean	SD
5–10	F	12	8.0	1.4	10.8	3.5	13.4	5.1	11.6	3.6	13.8	4.7
	M	15	6.4	1.2	8.6	1.9	10.2	2.5	9.5	1.8	10.6	2.2
10–15	F	9	12.4	1.4	19.0	5.3	21.3	8.0	20.6	5.0	23.2	8.9
	M	11	12.3	1.4	21.0	5.3	23.9	6.6	22.5	5.0	26.3	6.8
15–20	F	15	17.5	1.5	26.1	4.4	29.7	4.5	27.9	4.4	31.1	4.1
	M	10	18.1	1.5	45.2	6.2	47.3	7.4	46.9	8.6	48.4	9.7
20–30	F	32	25.6	2.9	26.5	4.9	30.9	6.0	29.2	5.1	34.1	6.2
	M	27	24.5	2.9	45.4	6.0	50.7	7.7	48.5	5.8	53.9	6.8
30–40	F	31	35.0	2.8	28.3	5.0	32.9	4.4	30.8	5.2	35.9	5.0
	M	32	35.4	3.0	42.1	7.9	46.7	8.5	45.8	8.6	50.3	9.1
40–50	F	32	45.3	3.1	27.9	4.4	33.0	5.1	28.9	4.4	33.6	4.4
	M	26	44.5	3.1	46.1	7.7	51.1	8.7	47.7	7.4	53.2	8.6
50–60	F	29	55.1	2.5	26.5	3.8	30.9	4.2	27.9	3.6	32.9	5.0
	M	11	54.0	3.4	42.5	7.3	47.9	7.6	46.5	6.4	51.1	8.1
60–70	F	21	64.8	2.9	23.1	4.2	26.7	5.0	24.6	4.7	27.9	5.6
	M	11	64.4	3.5	40.4	7.3	45.7	9.4	41.6	8.8	46.8	11.7
70–80	F	17	73.7	2.8	23.9	4.0	25.2	4.6	25.5	4.2	27.2	5.3
	M	5	74.5	2.9	37.4	4.2	40.6	3.8	38.4	3.8	42.8	6.7

### Test-retest reliability assessment

**Fig. 3:**
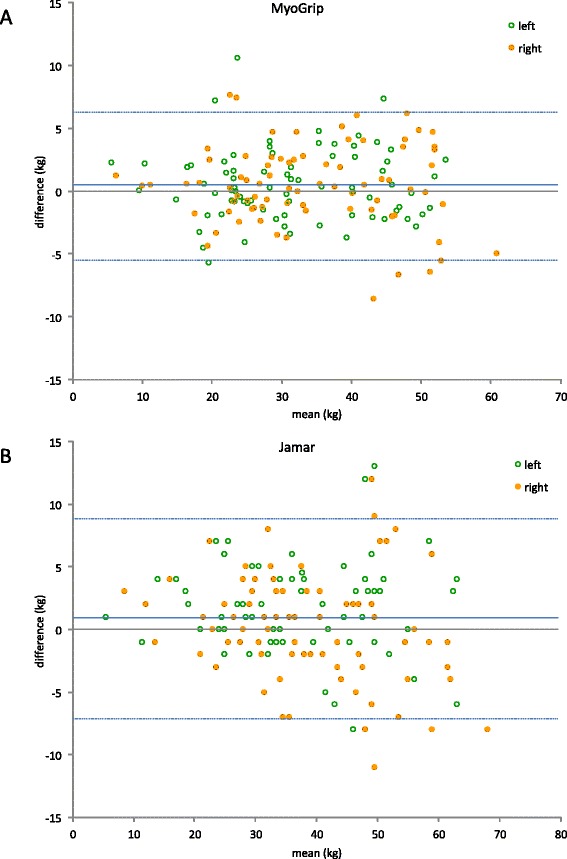
Bland & Altman plots for MyoGrip dynamometer (**a**) and Jamar dynamometer (**b**). Dotted lines represent the limit of agreements between measurements and can also be used to define the smallest detectable change

Seventy seven subjects returned for retesting. No rater effect was detected for either dynamometer. Table 3 details the results observed for test-retest reliability for both dynamometers. The results are shown as Bland and Altman plots in Fig. 3 for the MyoGrip and the Jamar dynamometers. Limits of agreement were slightly higher for the Jamar.

**Table 3:** Test-retest agreement and reliability

	MyoGrip	Jamar
Number of subjects	77	77
Mean difference (kg)	0.46	0.78
Absolute SEM (kg)	2.12	2.88
Relative SEM (%)	6.45	7.67
Upper limit of agreement (kg)	6.35	8.75
Lower limit of agreement (kg)	−5.42	−7.19
ICC [95 % CI]	0.967 [0.955;0.976]	0.947 [0.927;0.961]

### Correlation with the gold standard

**Fig. 4:**
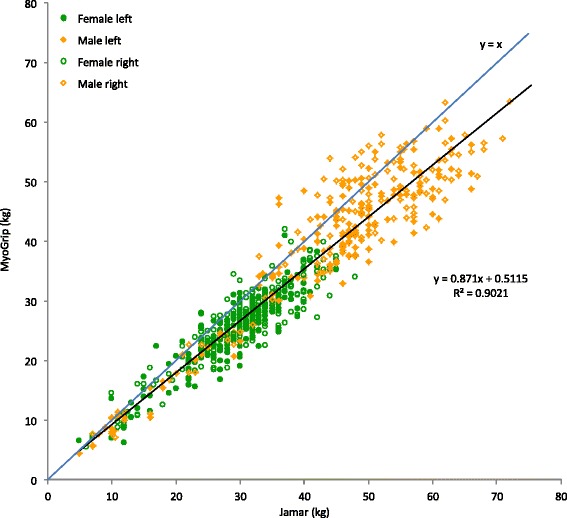
Relationship between grip strength measurements estimated with MyoGrip and Jamar. The line y = x represents the identity line (strict equivalence between measurements). The Jamar tends to reach higher grip strength estimates than the MyoGrip (approximately 14 % on average)

**Fig. 5:**
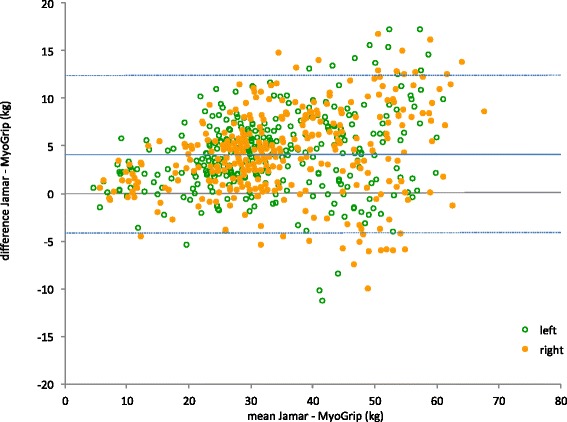
Bland & Altman plots for comparison between MyoGrip and Jamar. Dotted lines represent the limit of agreements between dynamometers

MGS obtained with the MyoGrip and the Jamar dynamometers were highly correlated (R = 0.950; p <0.0001) (Fig. 4). The Jamar tended to yield higher MGS estimate by 14.3 ± 13.8 % compared to the MyoGrip (p < 0.0001). This overestimation was not constant in absolute value but tended to increase when MGS increased (Fig. 5). Table 4 gives details on dynamometer agreement.

**Table 4:** Dynamometer agreement

	Jamar - MyoGrip
Number of subjects	346
Mean difference (kg)	4.12
Absolute SEM (kg)	2.98
Relative SEM (%)	8.79
Upper limit of agreement (kg)	12.37
Lower limit of agreement (kg)	−4.13
ICC [95 % CI]	0.900 [0.509;0.961]

### Predictive model

**Fig. 6:**
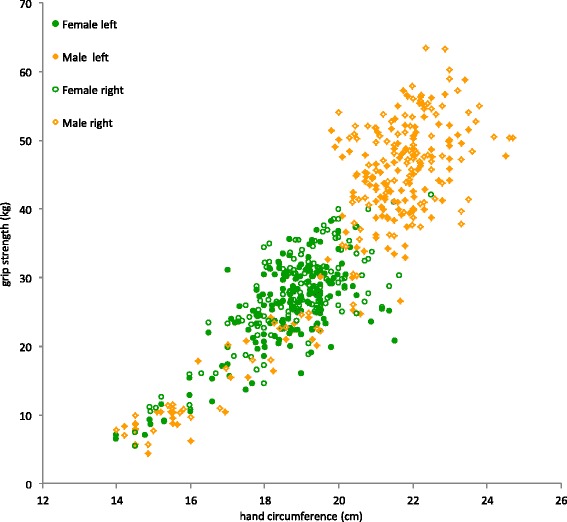
Relationship between grip strength and hand circumference. An excellent correlation was found between maximal grip strength and hand circumference (R = 0.885)

The predictive model was computed on subjects below than 60 years of age in order to avoid the possible effect of aging on the model parameters. A stepwise regression was first performed using the following variables: gender, age, height, weight, body mass index, fat mass percentage, hand length, hand circumference and forearm circumference. For both the MyoGrip and the Jamar dynamometers, the first variable chosen by the regression process was hand circumference as the main explanatory variable (Table 5). Fig. 6 illustrates the clear link between hand grip strength and hand circumference. Adding other variables did not add significant improvement to the regression-based model. Thus, hand circumference was used as a single variable and several models were tested (linear, quadratic, power, growth, exponential) in order to decide which was the best one. The best curve fit was obtained using a power equation.

**Table 5:** Results of the stepwise multiple regression (MyoGrip dynamometer) and simple correlations between maximal grip strength and variables

Variables in model	Correlation	F	Correlation	p
Intercept		974.9		
Hand circumference	0.885	2080.8	0.884	<0.001
Variables not in model	Partial correlation	F		
Sex	0.250	38.4	NA	
Age	−0.179	19.1	0.373	<0.001
Weight	−0.032	0.6	0.744	<0.001
Height	0.191	21.9	0.805	<0.001
BMI	−0.180	19.4	0.464	<0.001
% fat mass	−0.330	70.3	−0.188	<0.001
Forearm circumference	0.114	7.5	0.804	<0.001
Hand length	0.165	16.2	0.834	<0.001

For the MyoGrip, the equation is expressed as:

$$ \mathrm{M}\mathrm{G}\mathrm{S}\left(\mathrm{kg}\right) = 0.0003\ {\left({\mathrm{C}}_{\mathrm{hand}}\left(\mathrm{cm}\right)\right)}^{3.887}\left(\mathrm{adjusted}\ \mathrm{R}{}^2=0.825\right) $$

This equation can be simplified by a linear model without losing much relevance:

$$ \mathrm{M}\mathrm{G}\mathrm{S}\left(\mathrm{kg}\right) = 5.295\ {\mathrm{C}}_{\mathrm{hand}}\left(\mathrm{cm}\right)\ \hbox{-}\ 71.514\ \left(\mathrm{adjusted}\ \mathrm{R}{}^2=0.782\right) $$

For the Jamar, the power equation gives:

$$ \mathrm{M}\mathrm{G}\mathrm{S}\left(\mathrm{kg}\right) = 0.0006\ {\left({\mathrm{C}}_{\mathrm{hand}}\left(\mathrm{cm}\right)\right)}^{3.691}\left(\mathrm{adjusted}\ \mathrm{R}{}^2=0.781\right) $$

This equation can be simplified by a linear model:

$$ \mathrm{M}\mathrm{G}\mathrm{S}\left(\mathrm{kg}\right) = 5.554\ {\mathrm{C}}_{\mathrm{hand}}\left(\mathrm{cm}\right)\ \hbox{-}\ 72.294\ \left(\mathrm{adjusted}\ \mathrm{R}{}^2=0.740\right) $$

The principal application of the predictive model consists in computing the percentage of remaining strength of a given subject with respect to his/her theoretical value predicted by the regression-based model.

## Discussion

This study established normative data of maximal grip strength in healthy subjects aged from 5 to 80 years old and showed that maximal grip strength is highly dependent on hand circumference in children as well as in adults. A single regression-based predictive equation can be used for men and women and for children and adults. This may be highly useful when comparing individuals. The reasons and applications of such a relationship have already been discussed previously [20].

The development of a new dynamometer was motivated by the fact that none of the hand grip dynamometers on the market were adapted to very weak patients. Thus patients suffering from some disabling disorders such as Duchenne Muscular Dystrophy (DMD) or Spinal Muscular Atrophy (SMA) could not be reliably be evaluated as devices were not adapted to their weakness. Moreover, according to our own experience, the calibration of certain devices is not reliable and may be detrimental to the accuracy of the measure.

With regards to the Jamar dynamometer, MGS expressed by age range observed in the present study were consistent with the normative values observed in previous studies either in adults [18, 21–24] or in children [25, 26]. The right-handed subjects were significantly stronger on their right side, while no difference between sides could be detected in left-handed subjects. Brown et al. [27] already observed that left-handers were stronger on their right side. This can be interpreted for left-handers as an adaptation to an environment that is generally conceived by and for right-handers, since preference and performance may not be correlated [27]. As a limitation of the present norms, the age ranges are rather broad for children and adolescents as major changes may occur with increasing age and maturity. Unfortunately the number of participants was not statistically sufficient to further separate the subjects in smaller age ranges. Nonetheless predictive equations are a good replacement for norms.

These normative data have been obtained with an extended elbow position since Li et al. [28] have shown that elbow position (flexed at 90° or extended) has no influence on MGS estimation. Also according to physiotherapist involved in clinical trials in our centre, the extended position of the elbow allows less compensatory movements and better detection of compensations. However the extended elbow position cannot always be reached, for example in patients presenting with contractures such as in DMD.

Reliability of MGS measurement was very good with standard error of measurement approximately 6.5 % for the MyoGrip and 7.5 % for the Jamar. According to our experience, maximal isometric grip strength is one of the muscle functions that presents with lower coefficients of variation compared to other muscle functions, probably due to the fact that the effort is easily understandable, even in children.

An excellent correlation was observed between the values measured by the two dynamometers. However, MGS estimates were statistically lower when measured by the MyoGrip compared to the Jamar. The mean difference was about 14 %. Comparing the Grippit to the Jamar, Massy-Westropp et al. [15] observed that the Jamar yielded higher MGS by 22 N compared to the Grippit. Svantesson et al. [29] were not able to observe similar divergences. In the present study, the Jamar produced higher MGS estimate of 4.1 kg on average compared to the MyoGrip. Both dynamometers were calibrated before evaluations using static weights, whilst a MGS measure is rather an explosive application of force. Thus one explanation of the overestimation of force could be that the needle of the Jamar goes a bit higher than the actual strength due to its inertia.

MGS can be used as a good predictor of total muscle strength in healthy children, adolescents and young adults [30] and may be used as an overall health indicator in elderly people [31]. However an absolute strength estimate is meaningless without considering individual stature. For ageing application, this is particularly true due to the intergenerational stature difference illustrated by smaller body dimensions including hand. If not taken into account, one may tend to overestimate the muscle strength loss with age. Thus an absolute measure of strength is difficult to interpret solely because the stature of the subject greatly influences muscle strength [32]. Strength is roughly directly proportional to muscle mass and more specifically to the number of muscle fibres acting in parallel. In the absence of a reliable measure of muscle physiological cross-sectional area, an indicator of stature may be helpful to estimate what should be achieved with respect to the subject stature.

Relative values of MGS, expressed relatively to an index of stature, may help in better assessing the true loss of muscle quantity and/or quality. In the present study, hand circumference was found, as in a previous study in young adults [20], the best predictor of MGS. Indeed, various measurements of hand size have already been shown to be excellent indices of the whole body stature (for instance hand length [33]). Hand circumference seems to be a very good indicator of body stature, hence a good estimate of physical capacities. Hand size can be estimated using hand circumference (or hand width). Expressing MGS relative to an indicator of stature, such as hand circumference, may help in better situating individuals by minimizing the effect of their physical development as a confounding variable. It should be noted that the equations found in the present study are very close to those already described in a young adult population [20].

Assessing weakness using predictive models may by very practical in use in an aging population or in patients with neuromuscular disorders. Other applications are widespread such as rehabilitation, return to work or sport, surgical success as well as in monitoring the effects of training. Again, using relative grip strength estimates is a more robust method to assess muscle weakness; an absolute strength value is less meaningful because it obviously depends on the subject's stature.

## Conclusions

The MyoGrip is an innovative tool that has been designed for the assessment of very weak patients. Several studies have proved its usefulness and validity in DMD [34, 35] and SMA [36]. The present article proposes norms for this particular dynamometer, demonstrates its validity compared to the gold-standard and proves its reliability. Using hand circumference only, a single predictive equation for men and women, adults and children can be used to compute the predicted theoretical maximal grip strength. According to our knowledge, the metrological features of the MyoGrip dynamometer are currently unique. Such a device is useful for the detection of MGS when high precision and accuracy are required.

## Abbreviations

MGS: Maximal grip strength
CVar: Coefficient of variation
ICC: Intraclass correlation coefficient
SDD: Smallest detectable difference
SEM: Standard error of measurement
